# Monthly Report of Diseases

**Published:** 1801-01

**Authors:** 


### MONTHLY REPORT of DISEASES,

Admitted under the Care of the PHYSICIANS of the FINSBURY DISPENSARY, St. John's Square, Clerkenwell.

From November 20 to December 20, 1800.

| | No. of Cafes. | | No. of Cafes. |
|---|---|---|---|
| Continued Fever | 49 | Hypochondriafis | 2 |
| Small-Pox | 5 | Dyfpepfia | 8 |
| Eryfipelas | 2 | Gaftrodynia and Enterodynia | 8 |
| Cynanche Tonfillarum | 3 | Hyfteria | 4 |
| Pneumonia | 4 | Epilepfy | 3 |
| Hæmoptyfis | 2 | Vertigo | 2 |
| Phthifis Pulmonalis | 9 | Cephalæa | 5 |
| Cough and Dyfpnœa | 44 | Hemiplegia | 2 |
| Catarrh | 8 | Dropfy | 9 |
| Dyfentery | 1 | Afthenia | 25 |
| Diarrhœa | 4 | Difeafes of Infants | 14 |
| Chlorofis and Amenorrhœa | 10 | Chronic Eruptions | 10 |
| Menorrhagia | 6 | | |

From the above lift it will appear, that fevers of the typhoid character continue ftill to prevail, with fcarcely diminifhed violence, among the lower claffes in this metropolis.

For a very long period there has never occurred a feafon that has been characterized by fuch an univerfality and malignity of febrile contagion.

Many of the probable caufes of this peculiar prevalence of contagion have been ftated in more than one of our Reports; unfortunately, a principal of thefe caufes, the want of food, is likely to grow ftill more fatal and extenfive in its operation: what the circumftances are which may have induced this want, it is far out of the province of a Medical Writer to inquire; but it is within his province, and even it is his indifpenfable duty, to make known facts that are of the moft preffing importance, and that almoft exclufively fall within the fphere of his profeffional obfervation. It is a fact, and a moft miferable fact it is, that a large proportion of the difeafe with which the poor in London are afflicted, arifes from a deficient quantity of natural and wholefome nourifhment. To fuch feeble, hungry, and emaciated wretches, the adminiftration of drugs is farcical; it may be even regarded as cruel and infulting: to thofe who are afking for bread, it is giving a ftone. Not long fince, the Author of this Report was called to a family who, he was told, had been attacked by fever; but after examining the fkin, the tongue, the pulfe, the countenance, and the late hiftory of his poor patients, he foon found that their pitiable condition was entirely to be attributed to their not having taken food for three days. He beheld a child with his eyes open; but, as far as it could be afcertained from appearance, altogether infenfible. Not the fainteft veftige of fenfation, or power of motion, remained. His lower limbs had been for fome time in a ftate of contraction, but it was remarkable that this contraction was removed by expofing them for a few moments to the vivifying influence of the fire. His mother lying befide him on the fame miferable bed, although from a fimilar caufe herfelf unable to fpeak or move, every other moment caft a melancholy and anxious glance on the countenance of her famifhed and expiring child. The writer has been particularly induced to enlarge more perhaps than is confiftent with the proper bounds, and the general intention of this article, with regard to the prefent afflicting ftate of the difeafed poor, as a plan for their relief he underftands is at prefent in actual contemplation.

The nature of houfes of recovery has been explained, and the urgency of their importance has been already inforced in one of our medical reports. From that high fenfe of generofity, for which the inhabitants of this great capital are celebrated, almoft as much as for commercial induftry, it is beyond all doubt that they will be able and willing to carry into fpeedy and extenfive effect, an inftitution that will conduce more effentially to the good of the lower claffes of the community, and will redound more to the real honour of the higher, than perhaps any medical eftablifhment that has as yet been formed, or which even in future is likely to be conceived.

It is not amiable, nor is it reafonable to believe, that mankind are fo infenfible as fometimes they are reprefented to be; if they do not alleviate diftrefs, in general it is becaufe they do not *fee* it.

Let a perfon, however little diftinguifhed he may be for his kindnefs or humanity, accompany a phyfician in his melancholy round, among the fick poor, and he will feel his heart yearn for their mifery, and will find his hand open for their relief.

It is neceffary to remark, that in confequence of wanting the fupport of wholefome food, and the other unhappy circumftances of their condition, the poor in London are too frequently induced to feek a temporary relief from the ftimulating operation of the worft of fpirits.* Seldom poffeffing refolution of mind enough for the

* Such reflections as thefe, awakened by late fubjects of actual obfervation, may recal perhaps to the memory of the literary reader, fome melancholy paffages in the Hiftory and in the Letters of the Poetical Peafant of Scotland, both of which have been lately prefented to the public by the hand of a writer whofe the more ſpeedy modes of ſelf-deſtruction, they in general are driven to the ſlower ſuicide of habitual inebriety. By the more eaſy and opulent, theſe miſerable beings may ſometimes be accuſed of a criminal improvidence with regard to the future; but is it unnatural, or even is it unwiſe for thoſe not to look forward to the future, to whom the future preſents nothing but an unmixed proſpect of daily increaſing miſery and deſperation?

W. W.

*Red Lyon Square.* J. R.

---

### MEDICAL AND PHYSICAL INTELLIGENCE.

whoſe ſplendid and ſolid talents, with equal ſucceſs, have been employed in reſtoring the health of the living, and in embalming the memory of the dead. By ſome, Dr. Currie, in writing the life, may be ſuſpected of having thrown an undue luſtre on the character of the deceaſed Burns; but genius recently departed may be compared to the ſun, which never is obſerved to ſhine ſo beautifully as juſt after it has ſunk below the horizon.

